# Supplementary material for: All-at-once RNA folding with 3D motif prediction framed by evolutionary information
Source: Nat Methods. 2025 Oct 3;22(10):2094–106. doi: 10.1038/s41592-025-02833-w (PMC12510878; doi:10.1038/s41592-025-02833-w)
Supplement: Supplementary file 3 — Source code to run CaCoFold-R3D. Data and results. [file 41592_2025_2833_MOESM3_ESM.gz › supplemental_material/results/CaCoFold-R3D/CaCoFold-R3D_Rfam_vshuffle/RF02248_Six3os1_3.R2R.sto.pdf]

RF02248_Six3os1_3

5′ - A G A A U C ● R U ● U G G G G U C G G C Y R G C U C C G G A ● C U G C ● C R A G G U G G A C A C A C A A A A ● C A A A A G C C ○ A A G Y U G G R A A A
